# Stand Diversity Does Not Mitigate Increased Herbivory on Climate‐Matched Oaks in an Assisted Migration Experiment

**DOI:** 10.1111/pce.15383

**Published:** 2025-01-13

**Authors:** Juri A. Felix, Philip C. Stevenson, Nadia Barsoum, Julia Koricheva

**Affiliations:** ^1^ Department of Biological Sciences Royal Holloway University of London Egham UK; ^2^ Royal Botanic Gardens Kew UK; ^3^ Natural Resources Institute University of Greenwich Chatham UK; ^4^ Forest Research Farnham UK

**Keywords:** assisted migration, chemical defences, climate matching, monoculture, oak, plant–herbivore interactions, species mixture, tree diversity

## Abstract

Assisted migration is a tree‐planting method where tree species or populations are translocated with the aim of establishing more climate‐resilient forests. However, this might potentially increase the susceptibility of translocated trees to herbivory. Stand diversification through planting trees in species or genotypic mixtures may reduce the amount of damage by insect pests, but its effectiveness in mitigation of excess herbivory on climate‐matched trees has seldom been explored. Using the Climate Match Experiment which manipulates both tree climatic provenance and stand diversity, we compared growth, insect herbivory and leaf traits of pedunculate oaks (*Quercus robur*) of local and Italian provenances in monocultures, provenance mixtures or species mixtures. Additionally, we investigated whether tree apparency and light availability cause variation in leaf traits and herbivory and tested whether these factors were influenced by stand diversity. We found that Italian oaks were subject to greater herbivore damage than those of local English provenance regardless of stand diversity and that insect herbivory in Italian oaks was higher on more apparent trees. Italian oaks also had lower concentrations of hydrolysable tannins than English oaks, but tannin concentrations were poor predictors of herbivory. Additionally, we show that leaf trait variation is strongly associated with differences in light availability.

## Summary statement

This study explores the interactive effects of assisted migration and stand diversification on leaf traits and herbivory using a tree diversity experiment in Southern England. Oaks of Italian provenance received more herbivory than oaks of the local provenance, revealing a potential downside of assisted migration. Increasing neighbourhood diversity did not mitigate higher herbivory. Additionally, light availability significantly affected oak leaf traits; however, this did not explain variation in herbivory.

## 1 Introduction

Global forest restoration and tree‐planting schemes are helping to preserve biodiversity and mitigate CO_2_ emissions (Andres et al. 2023; Burton et al. 2018; Waring et al. 2020). However, these efforts are increasingly being undermined by poor tree growth and increased tree mortality resulting from the warming climate and the impact of invasive pests (Ammer 2019; Dawson et al. 2011; Millar and Stephenson 2015; Wessely et al. 2024). Approaches such as assisted migration and stand diversification have been proposed to minimise these threats (Barsoum 2015; Sacco et al. 2021), yet there has been little research on how these methods can be used in combination (but see Field et al. 2019).

Forests with potentially greater resilience towards future extreme temperatures and drought may be established through the use of assisted migration, which involves the targeted introduction of species or genotypes with specific climatic adaptations into forest ecosystems (Barsoum 2015; Broadmeadow, Ray, and Samuel 2005; Champagne et al. 2021; Duveneck and Scheller 2015; Xu and Prescott 2024). Many temperate tree species have wide geographic distributions (Eaton et al. 2016; Morin, Augspurger, and Chuine 2007); therefore, suitable tree genotypes can be sourced from specific locations which are matched to the expected future climatic conditions, referred to as ‘climate matching’ (Barsoum 2015; Broadmeadow, Ray, and Samuel 2005).

While ‘climate‐matched’ trees have adaptations that are predicted to make them more suitable to future climatic conditions (e.g., greater tolerance to drought stress), they may differ from local provenances in specific characteristics such as their leaf traits and consequently their relative susceptibility to herbivory (Champagne et al. 2021; Field et al. 2019; Sinclair et al. 2015). Genotypic variation and environmental adaptations could cause trees from different provenances to differ in their defensive traits and susceptibility to herbivores, which could be an important consideration when establishing resilient forests (Damestoy et al. 2019; Solla et al. 2016). Trees from lower latitudes are predicted to receive greater damage from pests and, therefore, may express higher levels of constitutive defences (Coley and Aide 1991; Schemske et al. 2009). This assumption has been challenged, however, by recent studies (Moles et al. 2011; Moreira et al. 2018; Poeydebat et al. 2020; Valdés‐Correcher et al. 2021).

Tree genotypes from lower latitudes have also been shown to express phenological adaptions more suited to warmer climates such as earlier budburst (Sinclair et al. 2015). In oaks, budburst is closely associated with maximum levels of defensive hydrolysable tannins (Salminen et al. 2004); therefore, early budburst in climate‐matched oaks could result in a mismatch between peak defences and intense springtime herbivory (Sampaio et al. 2016; Sinclair et al. 2015).

Stand diversification could potentially mitigate higher herbivory on non‐local provenances introduced through assisted migration, as it is well established that trees growing in species or genetically diverse neighbourhoods often suffer less damage from insect herbivores than those planted in monocultures (Guyot et al. 2015; Jactel, Moreira, and Castagneyrol 2021; Sacco et al. 2021; Tang et al. 2022). Alongside the increasing direct threats from high temperatures, droughts and extreme weather, climate change is expected to amplify the impact of insect pests over the coming decades (Pureswaran, Roques, and Battisti 2018; Seidl et al. 2018), emphasising the need to build forest resilience against both biotic and abiotic stress.

While high stand diversity is typically associated with lower insect herbivory on trees, this can be context‐dependent, with trees in diverse neighbourhoods sometimes experiencing more herbivory than those in monocultures (Berthelot et al. 2021; White and Whitham 2000). Several mechanisms can explain variation in diversity effects on plant–herbivore interactions (Barbosa et al. 2009; Root 1973; Stemmelen et al. 2022). For instance, according to the plant apparency theory, more apparent plants (e.g., trees larger than their neighbours) are more likely to be found by herbivores and thus may suffer greater rates of herbivory (Feeny 1976). Stand diversity may therefore influence herbivory rates by increasing or decreasing plant apparency due to the presence of larger or shorter neighbouring trees (Castagneyrol et al. 2013).

In addition, variation in physical and chemical leaf traits, which together are known to influence leaf quality, might drive reduced rates of herbivory in species mixtures (Awmack and Leather 2002; Endara, Forrister, and Coley 2023). However, while some studies have found that trees in species mixtures have less palatable leaves (e.g., due to increased concentrations of chemical defences) than those in monocultures, other studies have shown the opposite (Castagneyrol et al. 2019; Muiruri et al. 2019; Poeydebat et al. 2020; Rosado‐Sánchez et al. 2018). These conflicting results indicate that the effect of species richness on leaf traits is not necessarily uniform and that leaf trait variation in species mixtures could, in some cases, lead to increased rather than decreased rates of herbivory (Felix, Stevenson, and Koricheva 2023).

Plant diversity is thought to cause leaf trait variation indirectly by affecting environmental factors such as light intensity (Callaway, Pennings, and Richards 2003). Leaves in high‐light environments are often thicker to reduce water loss and have increased concentrations of secondary metabolites resulting from increased rates of photosynthesis, while shaded leaves may have higher specific leaf areas (SLAs) to increase light capture (Poorter et al. 2019; Rozendaal, Hurtado, and Poorter 2006). Light availability for focal trees in diverse stands may either increase or decrease depending on the height of the neighbours or as a result of canopy stratification effects and was recently reported by Williams et al. (2020) to account for up to 61% of leaf trait variation in a tree diversity experiment. New insights into the relationship between leaf traits and herbivory in diverse tree stands may therefore be gained by measuring light availability, helping to inform how stand diversification can protect against insect pests.

In this study, we examined the mechanisms of assisted migration effects on plant–herbivore interactions in the Climate Match tree diversity experiment in Kent, the United Kingdom. Specifically, we measured chewing insect herbivore damage alongside chemical and physical leaf traits of pedunculate oaks (*Quercus robur*) of local and Italian provenance. Additionally, by comparing herbivory and leaf traits between oaks growing in single‐provenance monocultures, provenance mixtures or species mixture plots, we assessed whether increased diversity resulted in leaf trait variation and associational resistance to herbivory. Finally, the mechanisms by which tree diversity might influence leaf traits and herbivory were examined by exploring the influences of light availability and tree apparency.

We aimed to answer the following questions:

- Do local and climate‐matched provenances of oak (*Q. robur*) differ in their growth, chemical and physical leaf traits and susceptibility to insect herbivory?
- Do tree growth, leaf traits and insect herbivory differ between single‐provenance monocultures, provenance mixtures and species mixture plots?
- Are the effects of tree diversity on leaf traits and insect herbivory mediated by changes in light availability and tree apparency?

## 2 Materials and Methods

### 2.1 Experimental Design

**Figure 1:**
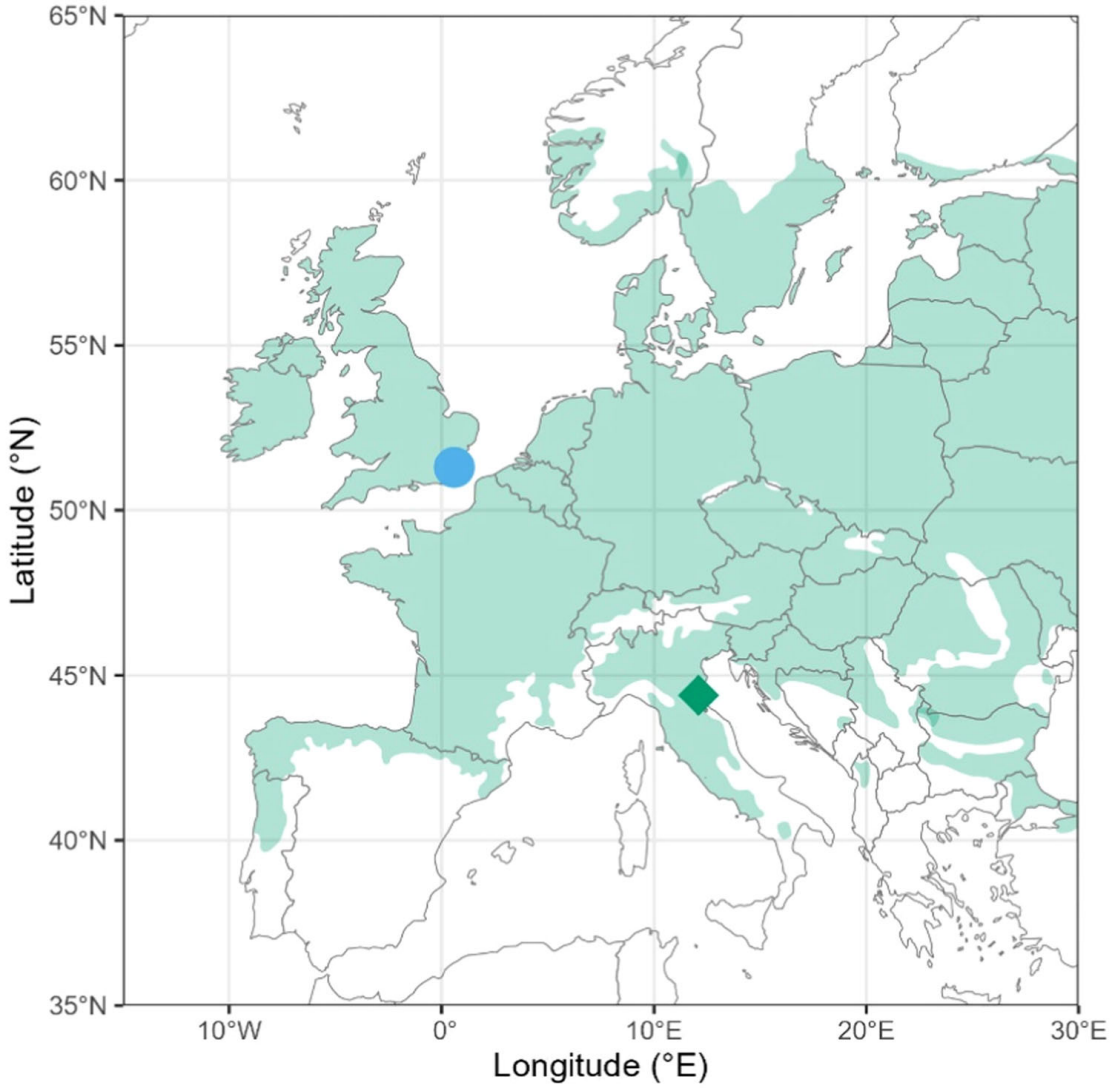
Map showing the location of the Kent experimental trial (blue circle) and the location of the tree nursery from which Italian provenance trees were sourced (green diamond). The shaded green area shows the European distribution of pedunculate oak (*Quercus robur*).

The Climate Match experimental trial at Hucking in Kent, South East England (51.29 N, 0.63 E), was established in 2011 (Barsoum 2015). The experimental trial was planted with 3‐year‐old saplings of European ash (*Fraxinus excelsior*), pedunculate oak (*Q. robur*), sweet chestnut (*Castanea sativa*) and wild cherry (*Prunus avium*). Trees of three provenances—local, French and Italian—were planted in the experiment, with French and Italian trees selected from nurseries in warmer and drier regions of continental Europe, which represent climatic conditions expected to match the climate of the United Kingdom by the years 2050 and 2080, respectively (Figure 1, Barsoum 2015). Sweet chestnut is an introduced species in the United Kingdom, so it was only represented by French and Italian provenances in the experiment. The entire experimental trial area is protected from mammalian herbivore damage by fencing and for several years following planting each tree had a protective vole guard.

**Figure 2:**
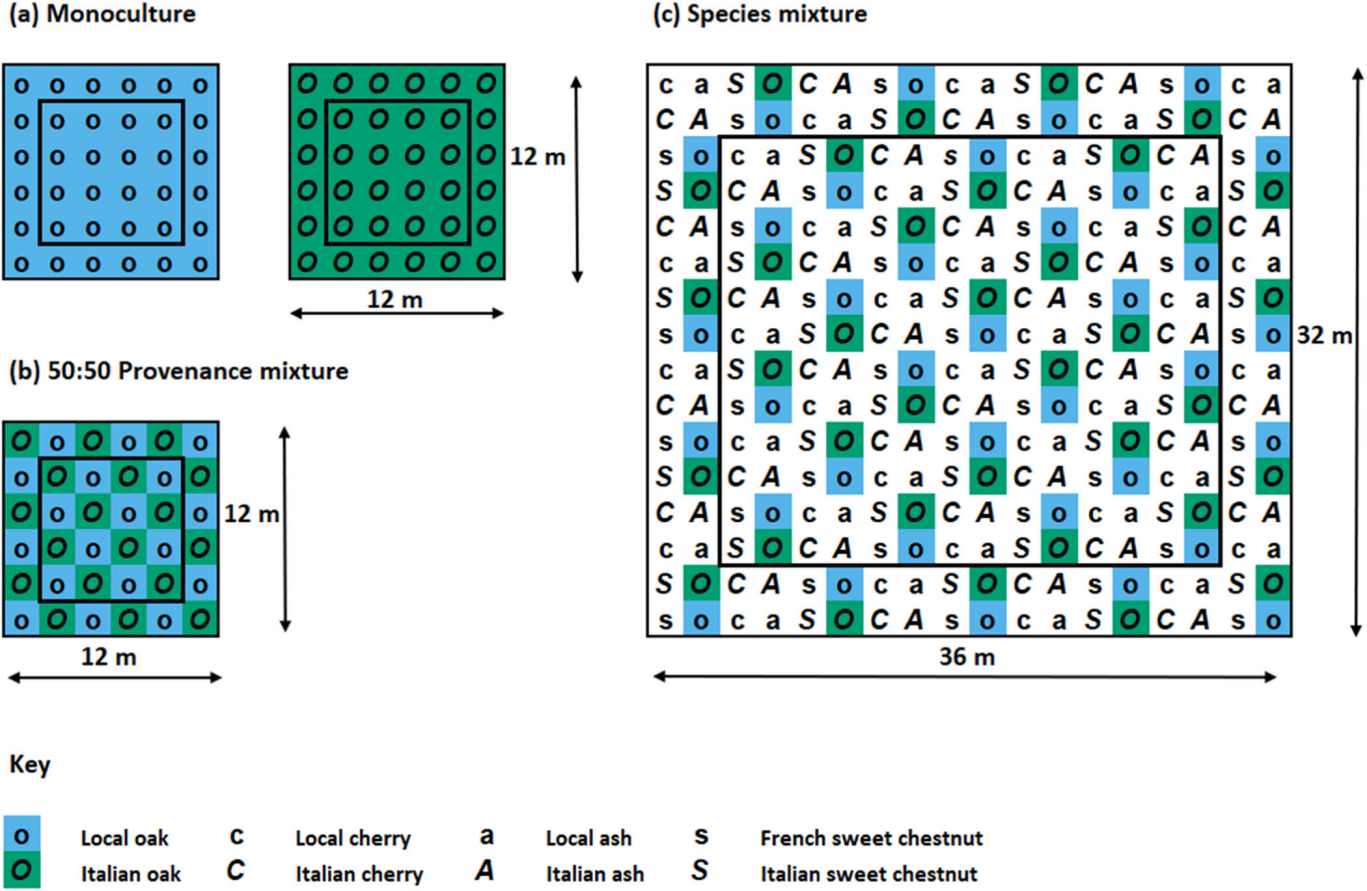
Layout of the three plot types used in this study: (a) monocultures of a single provenance (local or Italian), (b) 50:50 provenance mixtures containing trees of both local and Italian provenance, (c) species mixture plots, containing oak, cherry and ash trees from local and Italian provenances, and sweet chestnuts from French and Italian provenances. All ash trees growing within the experiment were either dead (~90%) or suffering from severe ash dieback symptoms (~10%). Trees were only sampled from within the inner black border of plots to minimise the influence of edge effects. [Color figure can be viewed at wileyonlinelibrary.com]

The experiment consists of trees planted 2 m apart in 12 × 12 m monoculture and provenance mixtures plots, as well as in 36 × 32 m species mixture plots containing local and Italian provenance trees of all four species (except sweet chestnut, which is instead represented by French and Italian trees; Figure 2). A regular planting pattern was used in all plots, which were replicated three times across adjacent blocks (Barsoum 2015).

The ash dieback fungus *Hymenoscyphus fraxineus* has been present in the United Kingdom since at least 2012 and is abundant in southeast England (Mitchell et al. 2014). By the time our fieldwork was conducted in the summer of 2021, ash dieback had caused a very high rate of mortality among the ash trees at the Kent experimental site, causing the near‐complete absence of ash within species mixture plots. As a result, species mixture plots only contained three species and tree density in these plots was often lower than in monoculture and provenance mixture plots.

### 2.2 Tree Selection

Fieldwork was conducted in late June to early July of 2021 when the short‐shoot leaves (i.e., leaves which had emerged during spring budburst rather than lammas shoots which only emerge in summer) on all oaks were fully expanded. Local and Italian oaks were sampled in monocultures, 50:50 provenance mixture plots and species mixture plots (Figure 2). French oaks were not used in this study due to their absence in species mixture plots. Five trees per provenance were randomly selected for sampling within each plot, avoiding the outmost trees to control for edge effects. When repeated for each of the three blocks, this gave a total of 90 sampled trees (with 45 trees of each provenance).

### 2.3 Tree Growth and Apparency Measurements

Height and circumference at breast height (~1.3 m) measurements were taken of each focal tree. Diameter at breast height (DBH) was calculated by dividing the circumference by π. The height of eight nearest neighbours to each focal tree was also measured, from which tree apparency values were calculated as follows (Castagneyrol et al. 2013):

$$\mathrm{Apparency}=\frac{1}{8}\times \sum_{i=1}^{8}\frac{\mathrm{Height focal tree}\;F-\mathrm{Height neighbour}\;i}{\;\mathrm{Average distance between focal tree and neighbours}}$$

If a neighbouring tree was dead or missing, its height was recorded as 0 m. Trees in all plot types had been planted in evenly spaced rows 2 m apart; therefore, the eight closest neighbours surrounding each focal tree were on average 2.41 m apart when diagonal neighbours were accounted for. A positive apparency value indicated that the focal tree was on average taller (more apparent) than its closest neighbours, and negative values indicate that it was on average shorter (less apparent).

### 2.4 Chewing Insect Herbivory Scoring

Four randomly chosen lower branches (1–1.5 m from the ground) on each focal tree were used for insect‐chewing herbivory assessments. Short‐shoot leaves (i.e., leaves which had emerged in the spring and rather than newly flushed lammas growth) were used for herbivory assessments and subsequent trait measurements as temporal variation in leaf traits and herbivory is lower for more mature leaves (Feeny 1970; Salminen et al. 2004). A total of 25 short‐shoot leaves were assessed from each branch and categorised into the following percentage categories of leaf‐chewing damage: 0%, 0.1%–5%, 5%–25%, 25%–50%, 50%–75% and 75+% (Muiruri et al. 2019). To calculate the average herbivory score for each branch, the midpoint of each damage category (e.g., 15% for the 5%–25% category) was multiplied by the number of leaves which fell into it and then divided by 25. The sum of these calculations for each damage category gave the herbivory score value for each branch, and the average of the four branches gave the herbivory score for each tree.

### 2.5 Leaf Trait and Light Measurements

Leaf thickness was measured using digital callipers on five undamaged short‐shoot leaves per tree from outer areas of branches at breast height. Taking care to select points in between the main veins, two thickness measurements were taken per leaf, from which average values were calculated.

Light was measured at four evenly spaced positions directly above sampled leaves around each focal tree using an LM‐50KL light meter (LATNEX, Toronto, Ontario). This was done between 10 a.m. and 2 p.m. on a clear day in mid‐July to reduce variation in light intensity between measurements. Light was measured in units of lux (range ≈ 2500–110 000 lux), which were then converted to units of photosynthetic photon flux density (µMol/m^2^/S) by dividing by the conversion factor of 54 (Thimijan and Heins 1983).

A total of 10 undamaged short‐shoot leaves were randomly sampled from the outer areas of several branches at breast height around each focal tree and were stored in a cool box until processing. A subset of 5 of the 10 leaves from each tree was photographed with a scale bar, and their leaf area was measured using the ImageJ software (Schneider et al. 2012). All leaves were then freeze‐dried (Krakowska‐Sieprawska et al. 2022), and the five leaves which were photographed were weighed so that SLA could be calculated by dividing the area of fresh leaves by their dry mass.

The 10 freeze‐dried leaves sampled from each tree were pooled together and ground to a fine powder using a pestle and mortar. A sample of leaf powder (10 mg) from each tree was then added to 1 mL of 70% methanol (10 mg/mL). Each sample was spiked with 0.1 mg/mL of reference compound chrysin which was used to quantify compounds. Solutions were left for 24 h and were then centrifuged to separate the leaf material from the supernatant. The resulting aliquots were transferred to 2‐mL vials and stored at −20°C until analysis.

The metabolite profiles of leaf extracts were collected using the ESI‐LC‐MS system, which consisted of an UltiMate 3000 Standard (SD) HPLC system (Thermo Scientific, the United States) coupled to a 100‐Hz diode array detector (DAD) and an Ion trap Velos Pro (Thermo Scientific) mass spectrometer. Chromatography was performed using a Luna C18 column (150 mm × 3 mm i.d., 3 μm, Phenomenex, the United States) at 30°C using a mobile phase gradient of 0:90:10 to 90:0:10 (acetonitrile:water:acetonitrile + 1% formic acid) over 20 min. 90% acetonitrile was held for 5 min and the mobile phase returned to initial conditions over 2 min, where it was held for a further 3 min (flow rate 400 μL/min).

Phenolic compounds were identified by comparing retention times, UV spectra and fragmentation patterns to chemical libraries and literature sources (Buche et al. 2021; Mady et al. 2023; Moilanen, Sinkkonen, and Salminen 2013; Molleman et al. 2024; Salminen et al. 2004; Tikkanen and Julkunen‐Tiitto 2003; Visakorpi et al. 2019). Molecular formulae were confirmed by comparing assigned formulae to those found using a separate high‐resolution LC‐MS system on which a small subset of oak leaves had been run.

Analysis of chemical data was performed using AnalyzerPro XD (SpectralWorks, the United Kingdom). Peak area values for all major peaks in negative ionisation mode were calculated in proportion to the reference compound chrysin, which was given a nominal peak area value of 1. This generated a list of peaks with average mass and retention time values and peak areas for all run samples. The phenolic compounds which were identified using LC‐MS as described above were then matched to this list, which gave the peak area for each identified phenolic compound in each leaf sample. Total phenolic abundance was calculated by summing peak areas of all identified phenolic compounds, while total flavonoids, condensed tannins and hydrolysable tannins were calculated by adding the peak areas of individual compounds assigned to each class. Due to a lack of reference compounds, individual peaks were putatively assigned.

### 2.6 Statistical Analysis

All statistical analyses were conducted in R version 4.2.2 (R Core Team 2021). Linear mixed models (LMMs) were run using the packages *lme4* (Bates et al. 2015) and *lmertest* (Kuznetsova, Brockhoff, and Christensen 2017). To determine the influence of provenance and stand diversity on tree growth, leaf traits and herbivory, LMMs were run with provenance (local or Italian), stand diversity (provenance monoculture, provenance mixture and species mixture) and their interaction as fixed factors and plot‐nested within block as a random factor. Post hoc Tukey tests were then run to investigate the pairwise differences between monocultures, provenance mixture and species mixtures using the package *emmeans* (Lenth et al. 2024).

Structural equation models (SEMs) were then constructed using the package *piecewiseSEM* (Lefcheck 2016) to explore whether the effects of stand diversity on herbivory were meditated by light availability, tree apparency and leaf traits and whether herbivory was correlated with stand diversity, apparency and leaf traits. SEMs were constructed separately for Italian and local oaks to investigate how relationships between factors differ between oaks of different provenance.

Measures of DBH and phenolic compound concentrations were square‐root transformed to meet assumptions of normality, while herbivory measurements were log‐transformed.

## 3 Results

### 3.1 Effects of Provenance and Stand Diversity on Tree Growth, Herbivory and Leaf Traits

**Figure 3:**
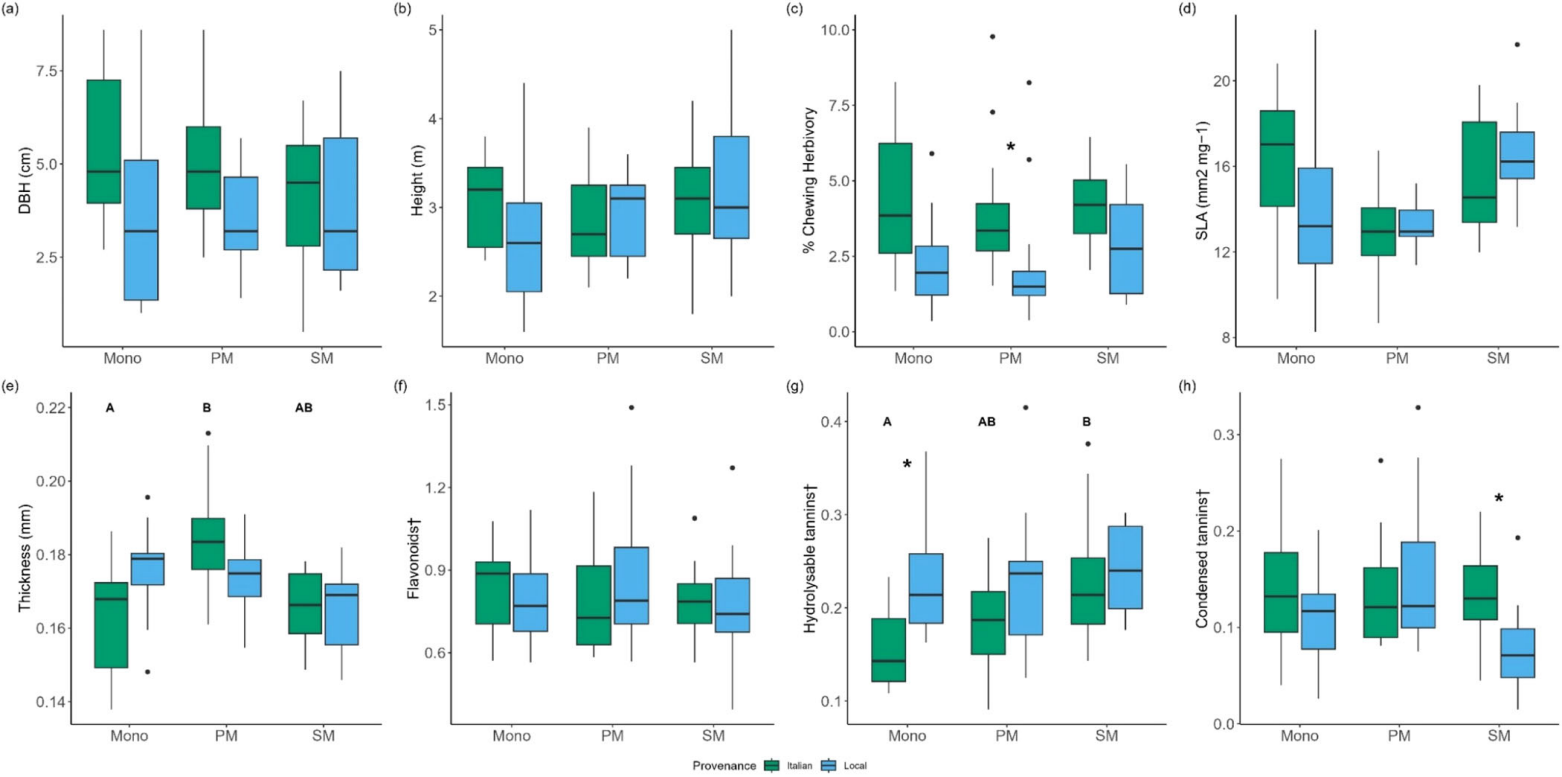
Tree growth (a, b), herbivory (c) and leaf traits (d–h) for Italian and local oaks growing in provenance monocultures (Mono), provenance mixtures (PM) and species mixture (SM) plots. †Phenolic compounds were measured as % peak area in proportion to chrysin which was present in all analytical samples at a concentration of 0.1 mg/mL. Results from post hoc tests are shown with different capital letters for significant differences between stand type and with asterisks for significant differences between provenances within the same stand type. [Color figure can be viewed at wileyonlinelibrary.com]

Italian oaks had a greater DBH than local oaks (4.86 ± 1.96 cm vs. 3.81 ± 2.05 cm, *p* = 0.018, Figure 3a), but the two provenances had similar height (Italian = 3.00 ± 0.56 m, local = 2.93 ± 0.76 m, *p* = 0.647, Figure 3b). Neither DBH nor height was affected by stand diversity (Table 1).

**Table 1:** Results from LMMs assessing the effects of provenance, stand diversity and their interaction on tree growth, leaf traits and rates of herbivory.

	Provenance			Stand diversity			Provenance × diversity		
	Df	*F* value	*p* value	Df	*F* value	*p* value	Df	*F* value	*p* value
DBH	1, 32.17	6.191	0.018*	2, 4.65	0.212	0.816	2, 21.27	1.460	0.255
Height	1, 30.48	0.213	0.647	2, 6.64	0.701	0.523	2, 22.94	0.710	0.390
SLA	1, 15.63	0.381	0.546	2, 6.98	1.513	0.284	2, 16.18	1.411	0.273
Thickness	1, 24.09	0.097	0.758	2, 4.21	4.286	***0.096***	2, 16.69	6.179	0.010**
Total phenolics†	1, 35.57	0.024	0.879	2, 4.10	0.379	0.706	2, 22.29	1.548	0.235
Flavonoids†	1.43	0.066	0.798	2, 6.08	0.320	0.738	2, 28.12	1.234	0.307
Hydrolysable tannins†	1, 84	14.347	< 0.001***	2, 84	4.439	0.0147*	2, 84	2.271	0.109
Condensed tannins†	1, 40.54	4.644	0.037*	2, 3.77	2.141	0.239	2, 23.87	3.916	0.034*
% Herbivory	1, 31.88	24.572	< 0.001***	2, 7.46	0.584	0.581	2, 24.71	0.223	0.802

*Note:* Significant p values are shown in bold with asterisks at the levels of “***” for *p* < 0.001, “**” for *p* < 0.01, and “*” for *p* < 0.05, and marginally significant p values (*p* = 0.05–0.1) are shown in bold italics. †Phenolic compounds were measured as % peak area in proportion to chrysin which was present in all analytical samples at a concentration of 0.1 mg/mL.

Chewing herbivory was significantly influenced by tree provenance and was almost twice as high on Italian oaks (mean ± SD = 4.68 ± 2.82%) as on local oaks (mean ± SD = 2.42 ± 1.78%). No overall effect of stand diversity on herbivory was found (Table 1); however, post hoc analysis showed that the difference in herbivory between local and Italian oaks was greatest in provenance mixture plots (Figure 3c).

A marginally significant effect of stand diversity on leaf thickness was found along with a significant interaction between stand diversity and provenance effects (Table 1). Post hoc analysis revealed that leaf thickness of Italian oaks was significantly lower in monocultures than in provenance mixtures, whereas stand diversity had no effect on the leaf thickness of local oaks (Figure 3e). SLA did not differ between the two oak provenances and was not affected by stand diversity.

A total of 50 phenolic compounds were identified in sampled leaves (Table S1). No qualitative differences in phenolic profiles were observed between local and Italian oaks as all identified phenolic compounds were present in oak leaves of both provenances. Moreover, neither total phenolics nor flavonoids were influenced by provenance, stand diversity or their interaction.

Local oaks were found to contain higher concentrations of hydrolysable tannins but lower concentrations of condensed tannins than Italian oaks (Table 1). Hydrolysable tannin concentrations were significantly influenced by stand diversity with Italian oaks in particular having lower concentrations of hydrolysable tannins in provenance monocultures than Italian oaks growing in species mixture plots (Figure 3g). For condensed tannins, there was a significant interaction between stand diversity and provenance effects (Table 1), and post hoc tests showed that Italian oaks contained higher concentrations of condensed tannins than local oaks only in species mixture plots (Figure 3h).

### 3.2 Structural Equation Model Analysis

**Figure 4:**
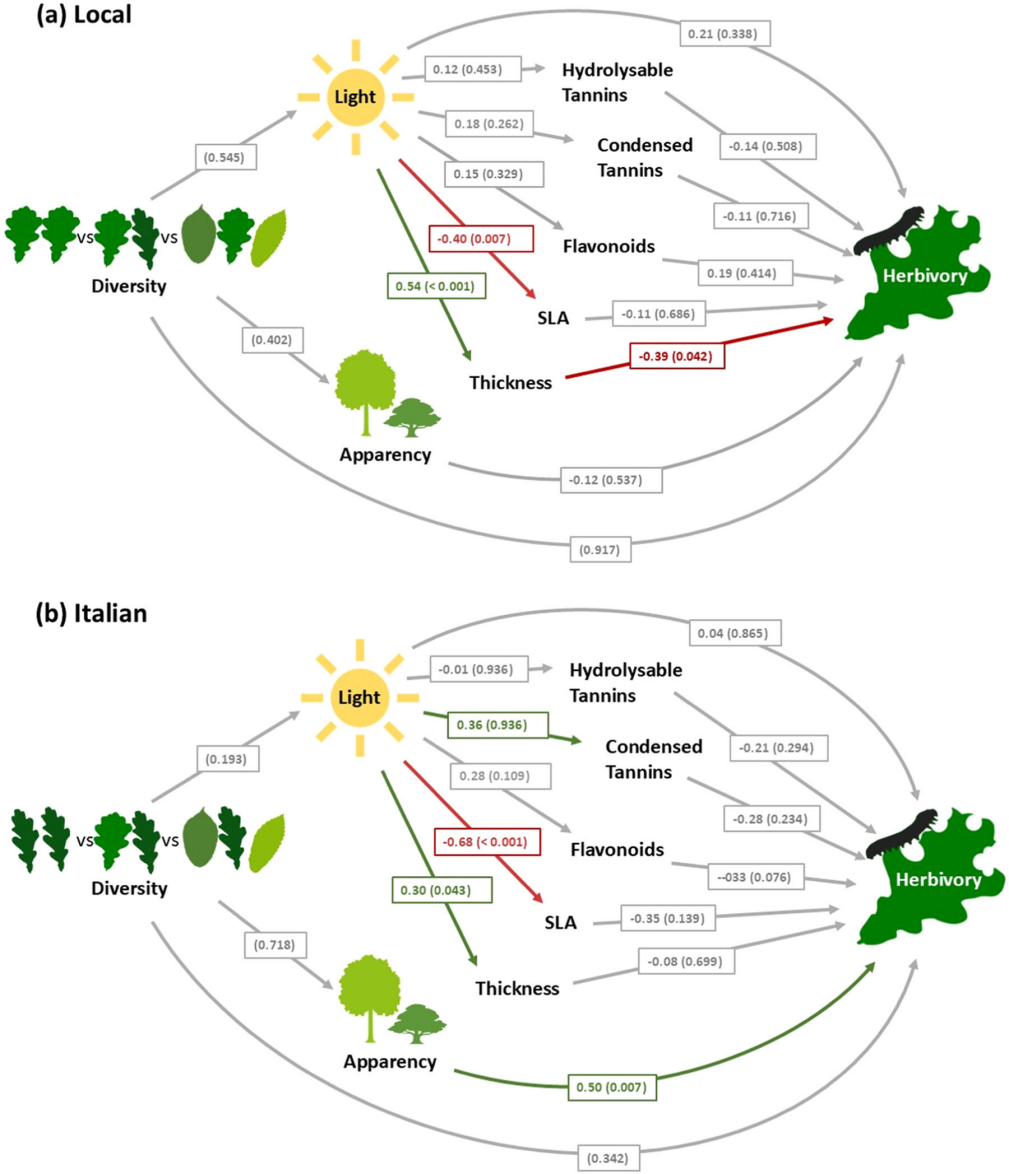
Structural equation models showing the relationships between stand diversity, light, leaf traits, tree apparency and herbivory for (a) local and (b) Italian oaks. Standard estimates of effects are shown for continuous variables with *p* values in brackets for both continuous and categorical variables. Arrows in green indicate significant positive effects, arrows in red indicate significant negative effects and arrows in grey show non‐significant relationships. [Color figure can be viewed at wileyonlinelibrary.com]

Structural equation models (SEMs) revealed a strong influence of light availability on oak leaf traits, with a significant positive effect on leaf thickness and a significant negative effect on SLA in both local and Italian oaks (Figure 4). Moreover, light availability had a significant positive effect on condensed tannins in Italian oaks but not in local oaks.

Although light availability varied greatly from tree to tree, it was not significantly influenced by stand diversity for either local or Italian oaks, with high‐ and low‐light environments recorded in all plot types. SEM analysis also confirmed that stand diversity had no direct effect on herbivory or tree apparency (Figure 4).

The only measured leaf trait that significantly influenced herbivory was leaf thickness, where thicker leaves had significantly lower herbivory, but only for local oaks (Figure 4a). No other leaf traits had significant relationships with herbivory in either local or Italian oaks, but herbivory in Italian oaks was positively associated with tree apparency (Figure 4b).

## 4 Discussion

We found that oaks of Italian provenance were subjected to higher damage from chewing‐insect herbivores than oaks of local English provenance when growing in a young tree diversity experiment. While this highlights a potential downside of planting climate‐matched trees, as even small increases of defoliation can slow rates of growth and carbon sequestration and decrease resilience to other forms of stress (Haavik and Stephen 2023; Visakorpi et al. 2021), the growth of Italian oaks did not appear to suffer, with average DBH values significantly greater and height similar to those of local oaks.

We also revealed provenance‐dependent effects of tree apparency on insect herbivory, and light intensity on leaf traits, indicating that provenance may also influence the strength of effects of external factors on plant–herbivore interactions. Neither tree apparency nor light intensity were significantly influenced by stand diversity, with similar leaf traits and herbivory rates recorded on Italian and local oaks similar within monocultures, provenance mixtures and species mixture plots. The lack of tree diversity effects suggests that excess herbivory on young climate‐matched oaks cannot be offset through stand diversification, although this may change over time as differences between stands intensify due to tree development and successional changes (Jucker et al. 2016).

### 4.1 Effects of Provenance on Insect Herbivory and Leaf Traits

As conspicuous, long‐lived species, oaks encounter sustained herbivory pressures throughout their lifespans and have evolved a suite of defensive traits including thick, tough leaves which contain several classes of defensive compounds such as flavonoids, hydrolysable tannins and condensed tannins (Feeny 1970; Forkner, Marquis, and Lill 2004; Moctezuma et al. 2014; Pearse 2011; Roslin and Salminen 2008). We investigated whether higher chewing‐insect herbivory experienced by Italian oaks could be explained by differences in phenolic compounds and physical leaf traits.

We found that the leaves of local oaks contained higher concentrations of hydrolysable tannins while the leaves of Italian oaks contained higher relative concentrations of condensed tannins. These two classes of tannins share common biosynthetic precursors; therefore, increased production of hydrolysable tannins may be at the expense of lower condensed tannin production and vice versa (Marsh et al. 2017; Salminen and Karonen 2011). There is some evidence that condensed tannins protect leaves against drought and UV‐B‐induced oxidative stresses (Close and McArthur 2002; Gourlay et al. 2022), which might explain why they were found at higher concentrations in Italian oaks.

We did not find a relationship between any measured chemical traits and chewing herbivory, implying that higher herbivory in Italian oaks is not due to lower concentrations of hydrolysable tannins. The effects of phenolic compounds on herbivores cannot easily be generalised and have been found in previous studies to depend on the concentrations of specific compounds, as well as herbivore specialisation, feeding guild and life stage (Roslin and Salminen 2008; Salminen and Karonen 2011). More nuanced effects of tannins and other phenolic compounds may therefore have been found if other categories of herbivory had been measured (e.g., leaf miners; Jia et al. 2023) and if the respective proportions of chewing damage due to different species could have been quantified (Moctezuma et al. 2014). Moreover, variation in other leaf traits which were not measured in this study such as leaf toughness, sugar content or foliar nitrogen concentration could have explained higher herbivory rates on Italian oaks (Awmack and Leather 2002; Pearse 2011).

Interestingly, SEM analysis revealed that predictors of herbivory differed between local and Italian oaks (Figure 4), with increasing leaf thickness correlated with lower herbivory in local oaks and greater tree apparency increasing herbivory in Italian oaks. This shows that the mechanisms governing plant–herbivore interactions can vary between tree provenances. Italian oaks, which were already more susceptible to herbivory than local oaks, received more herbivory when they were more apparent to herbivores and did not benefit from the protection that leaf thickness provides to more herbivore‐resistant local oaks.

### 4.2 Phenological Mechanisms

Leaf tannins in *Q. robur* follow a predictable shift across the growing season, with an increase in condensed tannins and a decrease in hydrolysable tannins from budburst in spring through summer to autumn (Feeny 1970; Salminen et al. 2004; Vanhakylä and Salminen 2023). The timing of budburst is a conserved trait within individual oaks (Crawley and Akhteruzzaman 1988) and is linked to provenance (Sampaio et al. 2016; Sinclair et al. 2015; Wright et al. 2021). Data collected at the Climate Match experiment in 2012 and 2013 (Barsoum, unpublished) shows that Italian oaks undergo budburst 7–10 days earlier than the local oaks, which could explain the relative differences in tannin concentrations between the two oak provenances.

Hydrolysable tannins are thought to cause oxidative stress in insect guts (Salminen and Karonen 2011), and their peak in concentration directly after budburst hypothesised to optimise defences against intense springtime herbivory (Barber and Fahey 2015; Feeny 1970; Visakorpi et al. 2019). Early budburst in Italian oaks could thus have resulted in a phenological mismatch between peak hydrolysable tannin concentrations and peak springtime herbivore abundance, leading to less well‐defended leaves at the time of most intense herbivory (Sinclair et al. 2015). Alternatively, Italian oaks may have suffered more herbivory because their leaves had been exposed to insect communities for a longer time period at the time of sampling (Barber and Fahey 2015; Sampaio et al. 2016).

Further fieldwork including continuous leaf trait measurements and herbivory assessments across the entire growing season is required to confirm whether phenological mismatches place climate‐matched oaks at increased risk of chewing herbivory.

### 4.3 Stand Diversity, Light and Apparency

We found that stand diversity exhibited a direct but limited effect on leaf traits, with Italian oaks expressing significantly higher leaf thickness in provenance mixture plots than in monoculture plots and higher concentrations of hydrolysable tannins in species mixture plots compared to monoculture plots. Niche‐partitioning effects may explain this effect of stand diversity; increased competition for water between drought‐resistant Italian oaks in monocultures could lead to increased leaf thickness as an adaptation to reduce transpiration rates (Gouveia and Freitas 2009), while increased nutrients available due to intraspecific variation in rooting depths in species mixture plots could increase investment into chemical defences (Liu et al. 2022). This explanation however fails to account for the lack of neighbourhood effects on local oaks.

By explicitly measuring light intensity, we found that light availability had consistent effects on leaf thickness and SLA, which were independent of stand diversity. Despite this, we found no direct link between light availability and herbivory, suggesting that light‐mediated leaf trait variation may only play a limited role in influencing chewing herbivory in oaks. In addition, we found a significant positive correlation between tree apparency and herbivory, although only for Italian oaks.

Inconsistent effects of stand diversity on oak leaf traits and herbivory have been reported in previous studies. For instance, Moreira, Glauser and Abdala‐Roberts (2017) found no effect of stand diversity on SLA and phenolic profiles but found a significant reduction in herbivory for oaks growing in species mixtures. In contrast, Castagneyrol et al. (2017) described an increase in SLA and a decrease in leaf toughness and thickness in oak species mixtures compared to monocultures but no variation in herbivory. Applying our findings on the influence of tree apparency and light availability reveals a pattern in previous research: oaks experience reductions of herbivory in species mixture when apparency is reduced due to taller neighbours (Castagneyrol et al. 2013; Moreira, Glauser, and Abdala‐Roberts 2017; Setiawan et al. 2016); and the variation of leaf traits is greatest between monocultures and species mixtures when there were large differences in light environments, either due to light‐blocking neighbours or because leaves were sampled at lower and more shaded branches (Castagneyrol et al. 2017, 2019; Galmán et al. 2022; Moore and Francis 1991). In short, differences in light availability and tree apparency appear to exert a more predictable influence on leaf traits and herbivory than stand diversity per se.

The effects of stand diversity on growing conditions and plant–herbivore interactions may only become noticeable in years or decades after stand establishment when trees have had considerable time to develop (Jucker et al. 2016). The lack of canopy closure due to the young age of the Climate Match experiment may thus explain why we failed to find a link between stand diversity, apparency and light availability in our study (Field et al. 2019). This may have been further compounded by ash dieback, which reduced tree density in the species mixture plots and opened large gaps in the canopy, thereby minimising the effects of taller neighbours around slow‐growing oak trees.

## 5 Directions for Future Work

Assisted migration could play an important role in buffering the negative effects of climate change on tree health, yet the selective introduction of new populations or species into different ecosystems remains a poorly understood and controversial strategy (Sinclair et al. 2015).

While we found that climate‐matched oaks experienced increased insect‐chewing herbivory, which could not be offset by stand diversification, the scope of our study was restricted to a single tree species during a specific growing season, which limits its applicability to other contexts. Moreover, only 90 trees were included in our experimental design, and it is possible that an expanded study incorporating data from more provenances, experimental sites or years may have yielded different results (e.g., Field et al. 2019). Further studies ideally including a wide range of species and provenances are needed to build a greater understanding of how assisted migration influences plant–herbivore interactions and other ecological processes.

The influence of stand diversity on plant–herbivore interactions was minimal in our study, likely due to the juvenile life stage of the trees. This provides useful insights into the limitations of stand diversity in buffering excess herbivory in recently established forest stands, which may be an important consideration during tree‐planting efforts. Further research using the Climate Match experiment in decades to come, when trees have matured and the effect of stand diversity has strengthened, may provide different findings and could provide vital insights into the interventions required to protect forests now and into the future.

## Conflicts of Interest

The authors declare no conflicts of interest.

## Supporting information

Supporting Information.

## Data Availability

The data and code used in this study are available on Zenodo (https://zenodo.org/records/14501208).
