# Supplementary material for: Stand Diversity Does Not Mitigate Increased Herbivory on Climate‐Matched Oaks in an Assisted Migration Experiment
Source: Plant Cell Environ. 2025 Jan 13;48(5):3620–31. doi: 10.1111/pce.15383 (PMC11963489; doi:10.1111/pce.15383)
Supplement: Supplementary file 1 — Supporting Information. [file PCE-48-3620-s001.docx]

| **Table S1:** List of phenolic compounds identified in oak leaves using LC-MS. *m/z* = mass/charge, RT = retention time (mins), MS2 = main fragmentation ions. | | | | | | |
| --- | --- | --- | --- | --- | --- | --- |
|  |  |  |  |  |  |  |
| **Assigned compound (or isomer)** | **Molecular Formula** | **Ion** | ***m/z*** | **RT** | **MS2** | **class** |
| Vescavaloninic acid | C_48_H_30_O_31_ | [M-2H]^2-^ | 550.15 | 2.31 | (1101), 528, 418, 359, 301 | hydrolysable tannin |
| Vescalagin or Castalagin | C_41_H_26_O_26_ | [M-2H]^2-^ | 466.39 | 2.32 | (933) 457, 435, 301, 275 | hydrolysable tannin |
| Pedunculagin | C_34_H_24_O_22_ | [M-H]^-^ | 783.19 | 2.34 | 765, 633, 481, 301, 275 | hydrolysable tannin |
| Procyanidin | C_30_H_26_O_13_ | [M-H]^-^ | 593.21 | 2.35 | 575, 467, 441, 425, 407, 303, 289 | condensed tannin |
| Vescavaloninic acid | C_48_H_30_O_31_ | [M-2H]^2-^ | 550.15 | 3.12 | (1101), 528, 418, 359, 301 | hydrolysable tannin |
| Vescalagin or Castalagin | C_41_H_26_O_26_ | [M-2H]^2-^ | 466.34 | 3.48 | (933) 457, 435, 301, 275 | hydrolysable tannin |
| Pedunculagin | C_34_H_24_O_22_ | [M-H]^-^ | 783.19 | 4.16 | 765, 633, 481, 301, 275 | hydrolysable tannin |
| Vescalagin or Castalagin | C_41_H_26_O_26_ | [M-2H]^2-^ | 466.29 | 4.35 | (933) 457, 435, 301, 275 | hydrolysable tannin |
| Procyanidin | C_30_H_26_O_13_ | [M-H]^-^ | 593.18 | 4.40 | 575, 467, 441, 425, 407, 303, 289 | condensed tannin |
| Pedunculagin | C_34_H_24_O_22_ | [M-2H]^2-^ | 783.09 | 4.90 | 765, 633, 481, 301, 275 | hydrolysable tannin |
| Procyanidin B (6)? | C_30_H_26_O_12_ | [M-H]^-^ | 577.25 | 4.92 | 559, 451, 425, 407, 299, 289 | condensed tannin |
| Procyanidin B6 | C_30_H_26_O_12_ | [M-H]^-^ | 577.29 | 5.35 | 559, 500, 451, 425, 407, 299, 289 | condensed tannin |
| Procyanidin C4 | C_45_H_38_O_18_ | [M-H]^-^ | 865.24 | 5.47 | 847, 739, 713, 695, 587, 575, 451, 425, 407, 287 | condensed tannin |
| Coumaroylquinic acid | C_16_H_18_O_8_ | [M-H]^-^ | 337.25 | 5.49 | 191, 163 | lignan |
| Catechin | C_15_H_14_O_6_ | [M-H]^-^ | 289.20 | 5.62 | 255, 175, 159 | flavonoid |
| 3-O-Feruloylquinic acid | C_17_H_20_O_9_ | [M-H]^-^ | 367.10 | 5.70 | 193, 134 | lignan |
| Procyanidin C4 | C_45_H_38_O_18_ | [M-H]^-^ | 865.27 | 6.25 | 847, 739, 713, 695, 587, 575, 451, 425, 407, 287 | condensed tannin |
| Punicalagin | C_48_H_28_O_30_ | [M-2H]^2-^ | 541.34 | 6.41 | (1083), 532, 510, 451, 425 | hydrolysable tannin |
| Procyanidin B6 | C_30_H_26_O_12_ | [M-H]^-^ | 577.21 | 6.55 | 559, 500, 451, 425, 407, 299, 289 | condensed tannin |
| Quercetin-3-O-arabinoglucoside | C_26_H_28_O_16_ | [M-H]^-^ | 595.26 | 6.61 | 475, 463, 445, 300, 271 | flavonoid |
| Myricetin-3-O-hexoside | C_21_H_20_O_13_ | [M-H]^-^ | 479.20 | 6.64 | 461, 433, 359, 316 | flavonoid |
| Eugeniin | C_41_H_30_O_26_ | [M-2H]^2-^ | 468.27 | 6.72 | (927) 458, 392, 383, 301, 169 | hydrolysable tannin |
| Punicalagin | C_48_H_28_O_30_ | [M-2H]^2-^ | 541.36 | 6.85 | (1083), 532, 510, 451, 425 | hydrolysable tannin |
| Quercetin galloyl hexoside | C_28_H_24_O_16_ | [M-H]^-^ | 615.21 | 6.97 | 463, 301 | flavonoid |
| Quercetin 3-O-β-D-glucopyranoside | C_21_H_20_O_12_ | [M-H]^-^ | 463.27 | 7.29 | 343, 301 | flavonoid |
| Ellagic acid | C_14_H_6_O_8_ | [M-H]^-^ | 301.00 | 7.39 | 271, 255, 229, 179, 151 | Phenolic acid |
| Isorhamnetin 3-O-β-D-glucopyranoside | C_22_H_22_O_12_ | [M-H]^-^ | 477.21 | 7.41 | 449, 357, 314, 285 | flavonoid |
| Quercetin-3-O-(acetyl)hexoside | C_23_H_22_O_13_ | [M-H]^-^ | 505.20 | 7.68 | 463, 445, 343, 301 | flavonoid |
| Kaempferol 3-(6''-galloylglucoside) | C_28_H_24_O_15_ | [M-H]^-^ | 599.23 | 7.68 | 463, 447, 313, 285 | flavonoid |
| Quercetin-3-O-pentoside | C_20_H_18_O_11_ | [M-H]^-^ | 433.08 | 7.75 | 300 | flavonoid |
| Isorhamnetin 3-(6''-galloylglucoside) | C_29_H_26_O_16_ | [M-H]^-^ | 629.18 | 7.77 | 477, 463, 313, 301 | flavonoid |
| Quercitrin | C_21_H_20_O_11_ | [M-H]^-^ | 447.26 | 7.85 | 327, 285, 255 | flavonoid |
| Isorhamnetin 3-O-β-D-glucopyranoside | C_22_H_22_O_12_ | [M-H]^-^ | 477.21 | 7.95 | 449, 357, 314, 285 | flavonoid |
| Kaempferol-3-O-hexuronide | C_21_H_18_O_12_ | [M-H]^-^ | 461.07 | 7.97 | 285 | flavonoid |
| Quercitrin | C_21_H_20_O_11_ | [M-H]^-^ | 447.26 | 7.97 | 327, 285, 255 | flavonoid |
| Isorhamnetin hexuronoside | C_22_H_20_O_13_ | [M-H]^-^ | 491.22 | 8.10 | 315 | flavonoid |
| Quercetin 3-(4''-acetylrhamnoside) | C_23_H_22_O_12_ | [M-H]^-^ | 489.29 | 8.35 | 313, 285 | flavonoid |
| pterocarinin A | C_46_H_36_O_30_ | [M-2H]^2-^ | 533.11 | 8.35 | (1067), 489 | hydrolysable tannin |
| Quercetin 3-alpha-L-arabinopyranosyl-(1->6)-(2''-(E)-p-coumaroylglucoside) | C_35_H_34_O_18_ | [M-H]^-^ | 741.37 | 8.37 | 695, 595, 505, 300 | flavonoid |
| Quercetin 3-(4''-acetylrhamnoside) | C_29_H_32_O_16_ | [M-H]^-^ | 489.20 | 8.95 | 429, 327, 299, 284, 255 | flavonoid |
| (+)-Gallocatechin-(4alpha->8)-(+)-catechin | C_30_H_26_O_13_ | [M-H]^-^ | 593.23 | 9.60 | 547, 447, 423, 307, 285 | condensed tannin |
| (+)-Gallocatechin-(4alpha->8)-(+)-catechin | C_30_H_26_O_13_ | [M-H]^-^ | 593.23 | 9.94 | 547, 447, 423, 307, 285 | condensed tannin |
| Kaempferol 3-(4''-acetyl-6''-p-coumarylglucoside) | C_32_H_28_O_14_ | [M-H]^-^ | 635.28 | 10.94 | 593, 575, 489, 471, 349, 285 | flavonoid |
| Kaempferol 3-(4''-acetyl-6''-p-coumarylglucoside) | C_32_H_28_O_14_ | [M-H]^-^ | 635.29 | 11.22 | 593, 575, 489, 471, 349, 285 | flavonoid |
| Kaempferol | C_15_H_10_O_6_ | [M-H]^-^ | 285.13 | 11.67 | 265, 241, 151 | flavonoid |
| Kaempferol 3-(2'',6''-di-(E)-p-coumarylglucoside) or isomer | C_39_H_32_O_15_ | [M-H]^-^ | 739.30 | 11.96 | 692, 633, 593, 575, 453, 307, 285 | flavonoid |
| Kaempferol acetyl dicoumaroyl hexoside | C_41_H_34_O_16_ | [M-H]^-^ | 781.37 | 13.09 | 763, 739, 709, 675, 575, 495, 349, 301, 285 | flavonoid |
| Kaempferol acetyl dicoumaroyl hexoside | C_41_H_34_O_16_ | [M-H]^-^ | 781.37 | 13.35 | 763, 739, 709, 675, 575, 495, 349, 301, 285 | flavonoid |
| Kaempferol diacetyl dicoumaroyl hexoside | C_43_H_36_O_17_ | [M-H]^-^ | 823.29 | 14.32 | 751, 677, 659, 617, 557, 537, 285 | flavonoid |
| Kaempferol diacetyl dicoumaroyl hexoside | C_43_H_36_O_17_ | [M-H]^-^ | 823.33 | 14.62 | 751, 677, 659, 617, 557, 537, 285 | flavonoid |
